# Smoking Pregnant Woman: Individual, Family, and Primary Healthcare Aspects

**DOI:** 10.3390/healthcare13091005

**Published:** 2025-04-27

**Authors:** Florina Ruta, Ion Mihai Georgescu, Geanina Moldovan, Laura Avram, Danusia Onisor, Zoltan Abram

**Affiliations:** 1 Department of Community Nutrition and Food Safety, George Emil Palade University of Medicine, Pharmacy, Science and Technology of Targu Mures, Gheorghe Marinescu Street No 38, 540136 Targu Mures, Romania; florina.ruta@umfst.ro; 2 Obstetrics Gynecology 1st Department, Braila County Emergency Hospital, 810325 Braila, Romania; 3 Department of Hygiene, George Emil Palade University of Medicine, Pharmacy, Science and Technology of Targu Mures, Gheorghe Marinescu Street No 38, 540136 Targu Mures, Romania; geanina.moldovan@umfst.ro (G.M.); zoltan.abram@umfst.ro (Z.A.); 4 Faculty of Economic Sciences, Dimitrie Cantemir University of Târgu-Mureș, Strada Bodoni Sándor 3–5, 540545 Targu Mures, Romania; 5 Department of Internal Medicine VII, George Emil Palade University of Medicine, Pharmacy, Science and Technology of Targu Mures, Gheorghe Marinescu Street No 38, 540136 Targu Mures, Romania; danusia.onisor@umfst.ro

**Keywords:** smoking during pregnancy, family environment, general practitioner

## Abstract

**Background:** Smoking during pregnancy continues to be a prevalent behavior in many countries, which requires smoking cessation intervention programs. **The aim** of this study was to investigate individual, family, and primary healthcare aspects that may influence continued smoking during pregnancy. **Methods:** This cross-sectional study involved 413 pregnant women registered with 50 General Practitioners (GPs) in Târgu Mureş County. Women voluntarily provided data to complete a questionnaire about socio-demographic, tobacco consumption, level of understanding of the risk of smoking during pregnancy, smoking in the family environment, and their own perception of the approach to smoking in primary healthcare. GPs facilitated the participation of patients in the study and offered logistical support for the data collection sessions held in their offices. **Results:** revealed that 49.39% of participants smoked before pregnancy, and 39.22% continued smoking during pregnancy. Continued smoking was significantly associated with having family members who smoked (OR = 8.83; 95% CI: 2.89–26.91) and a lack of anti-smoking informational materials at GP offices (OR = 5.68; 95% CI: 1.45–22.19). **Conclusion:** The findings highlight the critical role of family and primary healthcare interventions in smoking cessation during pregnancy. Therefore, tailored educational interventions at primary care settings are recommended.

## 1. Introduction

Smoking during pregnancy is a critical public health issue due to its significant impact on maternal and neonatal health, including increased risks of low birth weight, preterm birth, and higher rates of morbidity and mortality [1]. Among the risks associated with smoking are pre-eclampsia, placental abruption and placenta praevia, poor fetal outcomes such as premature birth, low birthweight, stillbirth, sudden infant death syndrome, and high overall perinatal mortality. Smoking during pregnancy was estimated at a prevalence value of 1.7% [2,3]. Despite extensive research and various interventions, smoking prevalence among pregnant women remains high, particularly in socioeconomically disadvantaged populations. Intervention models that led to increased smoking cessation rates during pregnancy were described, combining behavioral interventions and pharmacotherapy, behavioral counseling interventions provided through primary healthcare that included physician advice, nurse advice, individual counseling with a cessation specialist, group behavioral interventions, telephone counseling, mobile phone applications, providing informational materials on the effects of smoking on maternal and fetal health and the benefits of quitting as soon as possible on the fetus, providing incentives, and social support [4,5].

Smoking is currently considered as a threatening factor responsible for most of the health problems and deaths on a global level [6]. It is known that the life expectancy of a tobacco user decreases by 5 years with no occurrence of a health condition caused by smoking and by 15–20 years if the smoker develops smoking associated illnesses [7]. Quitting smoking should be approached as a medical service, and the recommendation to immediately give up smoking should be addressed to all tobacco users and be regarded as a duty by all members of the healthcare system [8,9].

The success in avoiding the risks of harming the mother’s and the infant’s health, through tobacco use during the pregnancy period, greatly depends on early interventions and elimination of the factors that contribute to the smoking continuation and the prevention of relapses [10].

The success of such approaches requires the understanding of the chronic nature of tobacco use as a disease, a commitment to long term monitoring and interventions in the acute stages of its manifestation, and the instruction of the medical staff taking care of the pregnant woman as well as of her family [11,12].

The monitoring of pregnant women by family practitioners is performed to identify which of them are or were active smokers or have smoking persons in their entourage. This identification, of women at risk, allows a targeted intervention that can be of great impact [13].

The dialogue about the harmful effects of smoking is performed in person (physician and patient). This way, the impact would be much greater, and the effects would be quantified quickly by quitting smoking. This dialogue could also be complemented by the distribution of leaflets presenting the negative role of smoking on the fetus [14].

Family medicine can provide opportunities to improve the clinical support for pregnant smokers. In this sense, it is necessary to simultaneously address the various specific barriers that prevent giving up tobacco use during pregnancy. The most important barriers to quitting smoking include poor knowledge and lack of patient confidence [15]. Currently, very few health professionals discuss with women how to support smoking cessation, both during pregnancy and postpartum. There is a gap between the understanding of these barriers and the measures to support at-risk groups and the way in which healthcare agencies and healthcare professionals can positively influence smoking cessation. Interpersonal and individual factors have an increased potential to influence how this support is provided [13,14]. Interpersonal and individual factors have an increased potential to influence how smoking cessation support is provided to young women of childbearing potential, and public health policy authorities should be aware of these factors [16].

A systematic review assessed the prevalence of smoking during pregnancy; the results showed marked differences between regions, with higher rates in low-income countries compared to high-income countries. These findings highlight the need for targeted and locally adapted interventions to reduce smoking prevalence among pregnant women and improve perinatal outcomes [3].

The barriers described by women to quitting smoking were not always fixed entities and depend largely on individual characteristics and social context. Sometimes, smoking was considered a necessary way of life to maintain mental stability and eliminate stress [17,18]. The group of barriers identified to quitting were the work environment, smoking habits of partners, and lack of knowledge of patients, while educational support, personalized counseling, and health information are factors that help to quit smoking [19].

Most clinicians working with pregnant women (family physicians, midwives, obstetricians) consider themselves responsible for assessing their patients’ smoking status [20]. However, data indicate that only about a third of professionals discuss with their patients options for support or treatment for quitting, and less than a quarter discuss smoking with women after the initial discussion [21].

Several studies have shown that professionals feel they lack the skills, confidence, motivation, and time to address and discuss smoking with their patients [22,23] Health professionals, particularly midwives, also express concerns that discussing smoking could negatively affect the therapeutic relationship between them and their patients [22,23]. However, few studies have investigated how the healthcare environment and health professionals can impact the approach to smoking in the antenatal period.

Other perceived barriers in addressing prenatal smoking are the lack of a coherent and unitarily coordinated multidisciplinary strategy and funding for its implementation [24].

This study aims to identify the individual aspects that favor the continuation of smoking during pregnancy, the socio-demographic factors as well as the level of understanding regarding the relationship between active smoking and the health risk for the mother and child among a group of pregnant women, the influence of the family environment on the pregnant woman in relation to smoking, and the evaluation of the anti-smoking intervention through primary healthcare through the personal perception of the pregnant women selected in the study.

The results will contribute to understanding the complexity of smoking behavior during pregnancy associated with socio-demographic and family factors as well as to understanding the possibilities of improving anti-smoking support from primary healthcare specialists.

## 2. Materials and Methods

### 2.1. Participants

This cross-sectional study involved pregnant women registered at family medicine clinics in Targu Mures. The study was conducted in 50 of the 90 registered family medicine practices in Mureș County. The selection of practices was based on the agreement of the family physicians to facilitate access to their patients.

**Figure 1:**
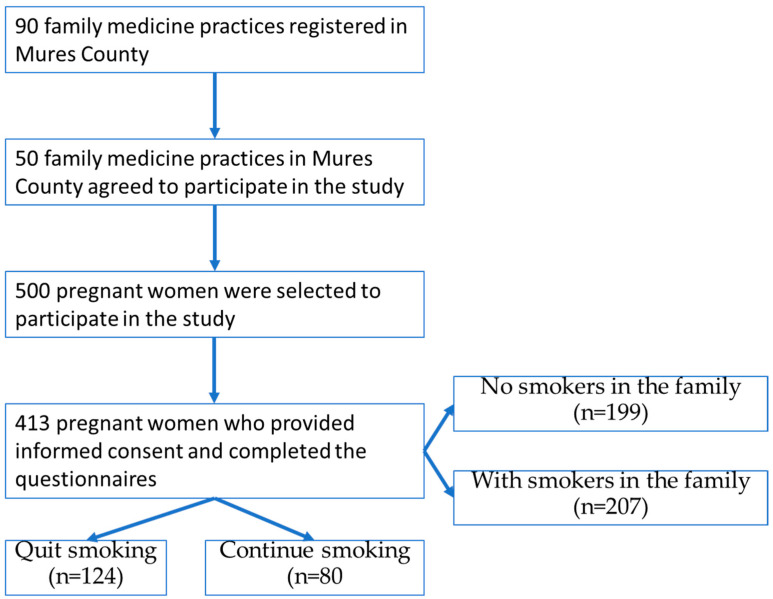
The design of our study.

The study included 413 women based on the following criteria: to be pregnant at the time of selection, to be patients of one of the family physicians in the selected practices, to benefit from informed consent to participate in the study, to be without known comorbidities at the time of enrollment in the study. The total number of pregnant women on the lists of participating family physicians was 500, of which 413 agreed to participate, resulting in a response rate of 82.6% (Figure 1).

The study included pregnant women who declared that they were smokers before pregnancy and those who declared that they were smokers at the time of selection. Exclusion criteria were the presence of certain diseases and the refusal of the patients to discuss their status regarding active or passive personal exposure to smoking.

Regarding Figure 1, it is inserted immediately after the inclusion criteria, without prior introduction or contextualization. The structure of the manuscript should be revised so that all figures and tables are correctly introduced and linked to the relevant content.

The steps established for conducting the study are presented in Figure 1.

The research protocol was approved by the Ethics Committee of the University of Medicine and Pharmacy Targu Mures as part of a larger study on building capacity for tobacco research in Romania, performed between 2013 and 2017. The survey response rate was 90%. We obtained written informed consent from our subjects, and all data were anonymous. All participants received comprehensive information about the study’s purpose, procedures, potential risks, and benefits. Written informed consent was obtained from every participant before their involvement in the study. To protect the identity of the participants, all personal identifying details were excluded from data collection instruments. Data were coded numerically, ensuring complete anonymity during analysis and reporting. Participation was entirely voluntary, with clear communication that participants had the right to refuse participation or withdraw at any stage without penalty or impact on their healthcare services. Given the sensitive nature of questions related to smoking habits during pregnancy, special care was taken to avoid any form of psychological distress or judgmental attitudes during interactions. Interviewers were trained to use neutral, supportive, and respectful language to minimize discomfort and facilitate open dialogue. Collected data were securely stored, accessible only by authorized research team members, ensuring confidentiality and protecting against unauthorized access or disclosure.

### 2.2. Research Methods

In order to evaluate the cessation of smoking intervention on pregnant women, we used a cross-sectional survey of 413 pregnant women, at the primary care level. The data collection instrument was a structured questionnaire specifically adapted for pregnant women. A comprehensive structured questionnaire consisting of 25 items was administered, which included the following: **Smoking status and behavior:** Questions regarding active smoking habits, passive smoke exposure, age at smoking initiation, smoking frequency, and intention to quit. **Socio-demographic data:** Information about age, education level, ethnicity, marital status, and residential setting (urban/rural). **Family environment:** Data on family smoking behavior, household smoking rules, and any behavioral changes in family members regarding smoking since pregnancy began. **Primary healthcare interactions:** Assessment of pregnant women’s observations and receipt of anti-smoking interventions during visits to GP offices, including presence and uptake of educational materials (brochures, leaflets). **Knowledge and awareness:** Items evaluating understanding of smoking-related health risks for both mother and child during pregnancy and postpartum.

These questionnaires were administered by trained research team members at GP practices during scheduled prenatal consultations, ensuring consistent and accurate data collection across all sites.

#### 2.2.1. Questions Regarding the Assessment of Tobacco Exposure

The main parameters taken into account were individual socio-demographic data (background, marital status, ethnicity, self-assessment of the level of knowledge regarding the risks of smoking and the advantages of quitting), smoking status (whether at the time of the questionnaire they were abstinent from smoking or whether they are active smokers), data on the family environment associated with smoking (exposure of the pregnant woman to passive smoking by family members), and the pregnant woman’s own perception of the extent to which she had access to informational materials about smoking through primary healthcare.

#### 2.2.2. Questions Regarding the Assessment of Anti-Smoking Interventions Provided by GPs

For example, the questions were as follows: “During the visit, did you observe informative material (leaflets, brochures) about nicotine consumption?”, “Smoking can cause complications with pregnancy and labor?”, “How many smokers are in the house where you live?”

#### 2.2.3. Statistical Analysis

For all the data in the database, we identified the number, percentage, and confidence interval (95% CI). For the questionnaire questions where participants had the option to choose a response (1 = disagree; …; 5 = agree), we calculated the mean, standard deviation (SD), and the result of the normality test, which led to the application of non-parametric tests, specifically the Mann–Whitney test. Even though we applied non-parametric tests, we chose to present the mean and standard deviation because the median values were not relevant (all were at their maximum values). Two multivariate logistic regression models have been used in order to evaluate the chances of continuing smoking compared to smoking cessation and smoking chances and if there are smokers in the family, including socio-demographic behavior and information received from the family doctor. Analyses were performed using the SPSS Statistics v22.0 statistical software. The significance level is 0.05.

## 3. Results

### 3.1. General Socio-Demographic, Family, and Primary Healthcare Aspects

The study involved pregnant women from 50 General Practitioners (GPs) who worked in different practices. A total of 25 family doctors worked in practices in Târgu Mureș, the county seat, while the other half were GPs in practices from other towns or communes in Mureș County. The average number of pregnant women registered per practice was 8.26. Family medicine practices in urban areas had a higher number of registered pregnant women compared to those in rural areas (urban 228 vs. rural 185), with differences also existing between the municipality of Târgu Mureș and other smaller towns in the county.

Out of 413 pregnant women interviewed, the mean age was 28.52 years old (SD = 5.61), and they were in an average pregnancy stage of 26 weeks (SD = 7.93, min = 6, max = 41) (189 patients were under 26 weeks of pregnancy vs. 217 over 26 weeks). In our study, 26.63% (110) of subjects had a low level of education (<10th grade), and 10.90% (45) were Roma ethnics. In this research, 44.79% (185) of the total studied female population live in rural areas, 48 of those continue to smoke, and 32 were not interested in quitting smoking. The low level of knowledge about smoking was identified in 17.43% (72) of women, and the indifference to passive smoking in 18.16% (75) of them. In our study 207 women (50.12%) have family relatives (including fathers) who smoke. (Table 1).

### 3.2. Socio-Demographic, Family, and Primary Healthcare Aspects in Relation to Smoking Status During Pregnancy

Table 2 presents the characteristics of pregnant women in Targu Mures, classified by smoking status and the number of smokers in the family (Table 2). Out of a total of 413 women, 124 quit smoking, while 80 continued to smoke. The average age was 28.5 years, and the average pregnancy stage was 25 weeks. Among them, 50.12% had family members who smoked, 26.63% had a low level of education, and 10.90% were of Roma ethnicity. A low level of knowledge about smoking was identified in 17.43% of women, and indifference to passive smoking in 18.16%. Smoking in the presence of pregnant women was reported by 7.99%, and the lack of informative materials at the family doctor was reported by 58.35%.

### 3.3. Individual Aspects Regarding the Pregnant Woman’s Understanding of the Relationship Between Exposure to Smoking and the Risk to the Health of the Mother and Child

The pregnant woman’s understanding of the health risk, as listed in Table 3, with reference to both the mother and the fetus, was significantly associated with smoking status during pregnancy.

The pregnant woman’s understanding of the health risk, as listed in Table 4, with reference to both the mother and the fetus, was significantly associated with the presence of smokers in the family environment.

### 3.4. Multifactorial Analysis Associated with Attitudes Towards Smoking During Pregnancy and the Risk of Passive Exposure Through Smoking Family Members

To investigate the complex interplay of factors contributing to smoking behaviors during pregnancy and passive exposure risks within family contexts, two multivariate logistic regression analyses were conducted. The first analysis compared pregnant women who continued smoking during pregnancy (n = 80) with those who successfully quit upon discovering their pregnancy (n = 124). The second analysis examined pregnant women exposed to smoking within their family environments (n = 207) versus those without family smokers (n = 199) (Table 5).

Presence of Smokers in the Family: Pregnant women with family members who smoke are significantly more likely to continue smoking during pregnancy (OR = 8.83; CI 95%: 2.89–26.91).

Low Level of Knowledge about Smoking: Pregnant women with low levels of knowledge about the risks of smoking are significantly more likely to continue smoking during pregnancy (OR = 12.61; 95% CI: 3.79–41.89).

Lack of Informative Materials: The presence of brochures or other educational materials at the family doctor’s office is associated with a higher likelihood of quitting smoking (OR = 5.68; 95% CI: 1.45–22.19) and receiving adequate support for this process.

## 4. Discussion

This study provides insight into the factors that may contribute to tobacco use and passive exposure among young women during pregnancy. A series of sociodemographic factors have the potential to influence tobacco use during pregnancy in women from an Eastern European country [25,26,27]. In these countries, some of which were formerly communist, the prevalence of smoking among women increased after the transition from centralized to market economies [28]. In Romania, the increase was dramatic, rising from 11% to 25% between 1989 and 2000 [29], and currently, our study data still indicate a significant prevalence of pregnant women actively and/or passively exposed to smoking, considering the maternal–fetal health risks associated with tobacco.

The analysis of the family environment showed that more than half of the women lived with smokers, with 34.4% having at least one smoker in the family. For 23.2% of respondents, smoking was allowed in certain areas inside the house, while 16.2% had no smoking restrictions in the house. For a quarter of those with smokers in the family, the habit of where to smoke did not change with the onset of pregnancy. On the day the questionnaire was administered, during the visit to the GPs’ office, more than half of the pregnant women did not notice and did not receive anti-smoking informational materials, and 14% of them stated that they were not asked if they were smokers.

Smoking in the family represents a significant public health challenge, considering the negative impact on non-smoking members, especially children and pregnant women [30]. Passive smoking exposure during pregnancy is associated with negative effects on pregnancy and the fetus. Non-smoking women, especially pregnant ones from low- and middle-income countries, are the most exposed, considering overcrowded households and uncontrolled smoking inside homes, leading to negative health consequences for women and their fetuses. Passive smoking is associated with low birth weight, preterm birth, stillbirth, small size for gestational age, and congenital malformations [30].

In our study, with an average age of 28.5 years, the percentage of continued smoking during pregnancy was 39%, and the percentage of pregnant women exposed to passive smoking was 7.99%. The number of patients less informed, through various means, by GPs regarding the risks of smoking and/or the benefits of quitting smoking, both for general health and specifically for maternal–fetal health, was higher among women who continued smoking during pregnancy and among pregnant women with smoking family members.

According to a study from the United Kingdom, interventions involving partners and family members were more successful in reducing smoking rates during pregnancy [31]. This finding is consistent with our results, which highlight the importance of involving the family in smoking cessation programs.

An important role in smoking cessation for pregnant women is related to anti-smoking interventions offered through primary healthcare. These interventions are essential for reducing the negative effects of smoking on the health of the mother and child. Family doctors, midwives, counselors, and other health professionals can provide education, counseling, motivational support, and financial incentives for smoking cessation among pregnant women [32]. Due to the insufficient use of pharmacological therapy for smoking cessation among pregnant women and the unclear balance between benefits and risks [33], behavioral interventions are expected to increase the chances of success [34].

The correct identification by respondents of the risks of smoking, consisting of pregnancy and labor complications, miscarriage, preterm birth, low birth weight, risk of asthma and frequent respiratory infections in childhood, and the health impact on the child from passive smoking exposure, was significantly associated with quitting smoking versus continuing smoking during pregnancy and the presence of smoking family members versus the absence of smoking family members [33].

The multifactorial nature of unhealthy behavior expressed through smoking shows the complexity of a problem that continues to be a threat in the population and is even more serious in vulnerable groups such as women and children. The irresponsible exposure of children, who are unable to protect their own health or make behavioral decisions, and women who lack protection in the family environment, highlights the need for a deeper understanding of the causes. The number of women exposed to passive cigarette smoking in their own homes is 2.2 times higher than that of men [34,35].

Our study highlighted that the factors most likely to influence attitudes towards smoking during pregnancy include being unmarried, a low level of understanding of the implications of smoking, exposure to passive smoking, and poor information about smoking through primary healthcare interventions.

Our study suggests that the family environment is a major factor influencing smoking behavior and can make quitting more difficult. It also highlights the importance of including the entire family in smoking cessation interventions, as having a smoker in the household can create an obstacle to quitting.

The low level of knowledge about smoking suggests the need for education about the risks of smoking, both for pregnant women and their partners or family members, to improve cessation outcomes. A recent study on digital health literacy also emphasizes the importance of educational interventions to improve health outcomes [36].

Our study also suggests the potential for using informational materials and involving family physicians in supporting pregnant women who want to quit smoking.

The results of this study will add to other international research that has explored the possibilities of assisting in smoking cessation, with potential extrapolation to vulnerable groups. For example, a study by Keten et al. (2023) [8] highlighted the significant role of social networks in supporting smoking cessation among pregnant women. Similarly, research conducted in the United Kingdom by Naughton et al. (2018) [17] emphasized the barriers and facilitators to smoking cessation from the perspective of health professionals. Derksen et al. (2021) identified a significant impact of social networks and found that social network support can facilitate smoking cessation during and after pregnancy [9]. These studies, along with our findings, underscore the importance of targeted interventions and the provision of informational materials by healthcare providers. Furthermore, a systematic review by Chamberlain et al. (2013) [12] on psychosocial interventions to support women in quitting smoking during pregnancy found that personalized support and educational materials significantly improve cessation rates. This is consistent with our observation that the lack of informational materials is a critical barrier to smoking cessation among pregnant women in Târgu Mureș.

In Romania, Brinzaniuc et al. (2018) [37] explored the socio-cultural context of smoking and cessation during pregnancy, finding that interventions targeting both the pregnant woman and her partner were more effective. Similarly, Bani-Yaghoub et al. (2018) [38] concluded that structured programs with clear guidelines and support materials were essential for success. A study on the effectiveness of structured programs in healthcare also supports these findings [39]. Comparing our results with these studies, we can conclude that the challenges and effective strategies for smoking cessation during pregnancy are consistent across different regions. This suggests that the interventions recommended in our study could be widely applicable and beneficial if implemented in similar contexts globally.

Additionally, family and financial stability were protective factors against tobacco use during pregnancy [39,40]. The low level of knowledge about smoking highlights the need to develop educational interventions based on formal computational models, designed to simulate and predict smoking-related behaviors among pregnant women and their families [41,42,43].

**Figure 2:**
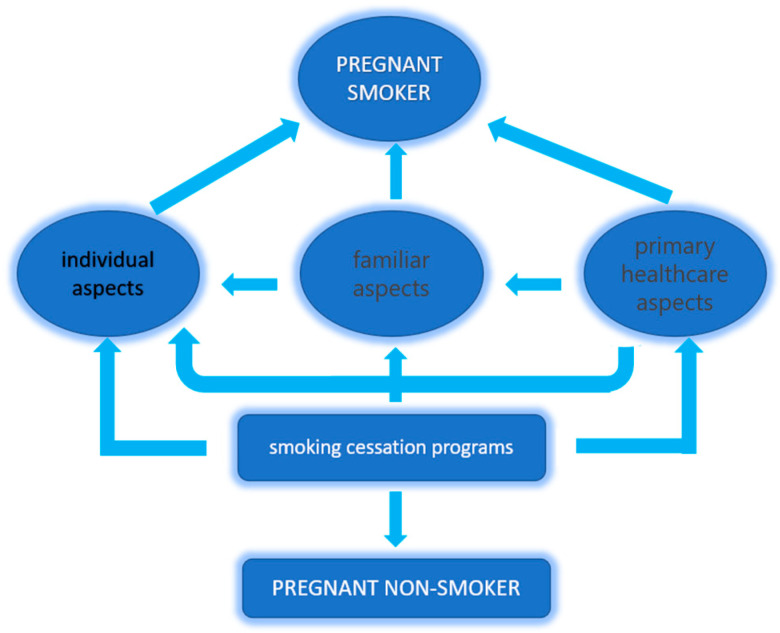
Simultaneous multifactorial approach to the risk of continued smoking during pregnancy.

This study contributes to the understanding of variables that can help reduce smoking habits among pregnant women, which need to be integrated alongside other health-promoting behavioral factors to improve the lifestyle of the general population and vulnerable groups in particular. The multifactorial involvement in maintaining an unhealthy habit like smoking requires a multipolar approach to combat it. Intervention strategies must effectively combine interventions targeting the vulnerable individual, the family environment with direct targeting of family members, healthcare personnel at all levels, and especially those at the primary healthcare level (Figure 2). Smoking cessation interventions should be better tailored and expanded, involving both family members and healthcare professionals, and should include accessible and targeted educational materials for pregnant women.

### Limitations

A limitation of this study is its reliance on self-reported data, which can introduce a social desirability bias. Pregnant women may underreport their smoking habits due to social pressures or a wish to present themselves more favorably, especially considering the health risks associated with smoking during pregnancy. This bias could result in an underestimation of the actual prevalence of smoking among participants. Future studies could address this issue by using biochemical validation methods, such as cotinine testing, to cross-check self-reported smoking status. In addition, the relatively small sample size in this study may limit and increase the likelihood of statistical errors. Despite this limitation, the findings offer valuable insights into factors affecting smoking behaviors during pregnancy, though caution is advised when interpreting results given the potential for underreporting.

Another limitation in data collection is the involvement of research team members who assisted participants in completing the questionnaires. This may have introduced interviewer bias, as participants’ responses could be affected by the interviewers’ presence or behavior. To reduce this risk, team members were trained in standardized, neutral questioning techniques and instructed to avoid any language that could introduce bias. Nonetheless, some degree of influence on responses may still be present.

## 5. Conclusions

Several barriers to smoking cessation during pregnancy were identified, including the pregnant woman’s family environment, insufficient anti-smoking interventions at the primary healthcare level, and a lack of awareness among the study participants regarding the maternal and fetal health risks associated with smoking. We believe that increasing smoking cessation rates among pregnant women requires a comprehensive approach that addresses multiple influencing factors simultaneously, rather than isolated measures targeting individual contributors to tobacco use. A critical factor to consider is the family environment, where the pregnant woman must be safeguarded from passive smoke exposure and actively supported in her efforts to quit smoking.

Effective anti-smoking interventions should integrate education for young women, both those preparing for pregnancy and those already expecting, alongside training for primary healthcare providers, who play a key role in direct interventions with pregnant women and their families. Survey of tobacco use and smoke exposure should be a routine part of the prenatal visit, and clinicians should provide pregnancy-specific counseling to those who smoke.

By ensuring a multi-faceted strategy, we can create a more supportive framework for smoking cessation during pregnancy.

## Figures and Tables

**Table 1:** Description of the studied group: individual aspects, family environment, primary healthcare.

Statement	n	%	CI 95%
**Individual aspects**			
Residence Urban Rural			
	228	55.2	50.4–60
	185	44.8	40.0–49.6
Marital status: Married Concubinage Unmarried Divorced			
	289	70.0	65.4–74.1
	62	15.0	11.6–18.4
	54	13.1	9.9–16.2
	8	1.9	0.7–3.4
Ethnicity Romania Hungarian Roma Other			
	276	66.8	62.5–71.2
	91	22.0	18.2–25.9
	45	10.9	8.0–14.0
	1	0.2	0.0–1.0
**Family Aspects**			
How many smokers are in the house where you live? 0 people 1 person 2 people More than 2 No answer			
		
	199	48.2	43.1–53.3
	142	34.4	29.8–39.2
	49	11.9	8.5–15.2
	16	3.9	2.2–6.0
	7	1.7	0.5–3.1
How is smoking seen in the place where you live? No one smokes where they live—they smoke outside Smoking is only allowed in certain rooms in the room Smokers can smoke anywhere they want No answer			
		
	245	59.3	54.2–63.9
		
	96	23.2	19.4–27.4
		
	67	16.2	12.6–20.1
		
	5	1.2	0.2–2.2
If there are smokers in the family, have they changed this habit since the pregnancy was highlighted? No changes were noted No one smokes in the house since I am pregnant—Smokes outside People smoke in other rooms when they find out they are pregnant People smoke anywhere in the house, even if they find out they are pregnant No answer			
		
		
		
	101	24.5	20.3–28.3
	176	42.6	37.8–47.5
		
		
	56	13.6	10.4–16.9
		
		
	33	8.0	5.3–10.7
		
		
	47	11.4	8.5–14.3
**Primary Healthcare Aspects**			
During today’s visit, did you observe any information materials (leaflets, brochures) on nicotine consumption? Yes No No answer			
		
		
		
	158	38.3	34.1–42.9
	241	58.4	53.8–62.7
	14	3.4	1.7–5.1
During today’s visit, were you asked by your doctor/nurse/other nurse if you were smoking? Yes No No answer			
		
		
		
	350	84.7	81.1–87.9
	58	14.0	10.7–17.7
	5	1.2	0.2–2.4
During today’s visit, did you pick up any informative materials that refer to cigarette smoking during pregnancy? Yes No No answer			
		
		
		
	98	23.7	19.6–27.8
	245	59.3	54.2–63.9
	70	16.9	13.6–20.6

**Table 2:** Smoking status during pregnancy associated with potential contributing factors.

Factors of Influence over the Abandonment Decision	Total (n = 413)	Quit Smoking (n = 124)	Continue Smoking (n = 80)	No Smokers in the Family (n = 199)	With Smokers in the Family (n = 207)
**Individual apects**					
Age (average, SD)	28.5 (5.61)	28.9 (4.95)	28.3 (5.81)	29.4 (4.94)	27.3 (6.00)
Pregnancy week (mean, SD)	25 (7.93)	25.0 (8.27)	26.6 (5.97)	24.8 (8.47)	26.6 (7.13)
Low study level	110 (26.63%)	30 (24.19%)	35 (43.75%)	35 (17.59%)	74 (35.75%)
Roma ethnics	45 (10.90%)	6 (4.84%)	19 (23.75%)	7 (3.52%)	38 (18.36%)
Rural residency	185 (44.79%)	45 (36.29%)	48 (60.00%)	67 (33.67%)	116 (56.04%)
Not married	124 (30.02%)	35 (28.23%)	38 (47.50%)	31 (15.58%)	91 (43.96%)
Low level of information on smoking	72 (17.43%)	32 (25.81%)	36 (45.00%)	15 (7.54%)	57 (27.54%)
Indifference to passive smoking	75 (18.16%)	21 (16.94%)	23 (28.75%)	24 (12.06%)	50 (24.15%)
**Family aspects**					
Presence of smokers in the house	207 (50.12%)	60 (48.39%)	74 (92.50%)	-	-
Smoking in the presence of pregnant women	33 (7.99%)	6 (4.84%)	16 (20.00%)	2 (1.01%)	31 (14.98%)
**Primary Healthcare Aspects**					
Lack of informative materials to the family doctor	241 (58.35%)	75 (60.48%)	57 (71.25%)	101 (50.75%)	137 (66.18%)
Lack of informative leaflets received from the family doctor	245 (59.32%)	70 (56.45%)	61 (76.25%)	104 (52.26%)	137 (66.18%)
Without informational materials such as brochures or leaflets about smoking during the visit to the GPs	176 (42.62%)	55 (44.35%)	54 (67.50%)	63 (31.66%)	112 (54.11%)

**Table 3:** Smoking status and knowledge of the effects of smoking on pregnancy and birth.

Statement	Quit Smoking (n = 124)	Continue Smoking (n = 80)	*p* Value
	Mean (SD)	Mean (SD)	
Smoking can cause complications with pregnancy and childbirth labor	4.631 (0.774)	3.663 (1.211)	<0.0001
Smoking during pregnancy can increase the risk of miscarriage	4.537 (0.958)	3.650 (1.170)	<0.0001
Smoking during pregnancy can increase the risk of a premature birth	4.537 (0.837)	3.696 (1.170)	<0.0001
Smoking during pregnancy increases the chances of having a low birth weight baby	4.439 (1.080)	3.625 (1.257)	<0.0001
Children of smokers are more likely to have asthma or any other respiratory infection	4.504 (0.927)	3.638 (1.255)	<0.0001
If my life partner consumes tobacco around me, it will increase the risk of pregnancy complications	4.233 (1.151)	3.354 (1.350)	<0.0001
If my life partner or other family members smoke around me, the chances of the child’s illness increase.	4.279 (1.093)	3.300 (1.409)	<0.0001

**Table 4:** Smoking family members and knowledge of the effects of smoking on pregnancy and birth.

Statement	With Smokers in the House (207)	Without Smokers in the House (199)	*p* Value
	Mean (SD)	Mean (SD)	
Smoking can cause complications with pregnancy and childbirth labor	4.063 (1.168)	4.656 (0.902)	<0.0001
Smoking during pregnancy can increase the risk of miscarriage	3.995 (1.193)	4.710 (0.822)	<0.0001
Smoking during pregnancy can increase the risk of a premature birth	4.059 (1.129)	4.691 (0.784)	<0.0001
Smoking during pregnancy increases the chances of having a low birth weight baby	3.980 (1.217)	4.591 (0.981)	<0.0001
Children of smokers are more likely to have asthma or any other respiratory infection	4.00 (1.150)	4.635 (0.934)	<0.0001
If my life partner consumes tobacco around me, it will increase the risk of pregnancy complications	3.730 (1.298)	4.460 (1.039)	<0.0001
If my life partner or other family members smoke around me, the chances of the child’s illness increase.	3.727 (1.311)	4.536 (0.981)	<0.0001

**Table 5:** Factors associated with continuous smoking (smoking cessation vs. follow-up) and smokers vs. no family smokers among pregnant women in Romania.

Variable	Quit Smoking (n = 124) vs. Continua (n = 80)			Non-Smokers (n = 199) vs. Smokers (n = 207) in the House		
	Odds Ratio	95% CI	*p*	Odds Ratio	95% CI	*p*
**Individual apects**						
Low study level	0.66	0.21–2.08	0.480	1.32	0.56–3.11	0.522
Roma ethnics	2.01	0.39–10.34	0.399	0.96	0.28–3.23	0.956
Rural residency	0.88	0.32–2.43	0.812	0.97	0.48–1.92	0.931
Not married	0.70	0.29–1.69	0.434	2.19	1.11–4.31	0.023 *
Low level of knowledge on smoking	12.61	3.79–41.89	<0.001 *	3.64	1.47–9.02	0.005 *
Indifference to passive smoking	0.49	0.17–1.39	0.183	1.28	0.56–2.92	0.547
**Family aspects**						
The presence of smokers in the house	8.83	2.89–26.91	<0.001 *			
Smoking in the presence of pregnant women	2.86	0.79–10.31	0.806	4.60	0.96–22.05	0.056 *

Lack of informative materials to the family doctor	0.23	0.06–0.83	0.025 *	1.10	0.50–2.40	0.804
Lack of informative leaflets received from the family doctor	5.68	1.45–22.19	0.012 *	1.31	0.56–3.01	0.524
Without informational materials such as brochures or leaflets about smoking during the visit to the GPs	1.96	0.76–5.05	0.161	2.23	1.13–4.43	0.020 *

CI, confidence interval. * Statistical significance.

## Data Availability

The data that support the findings of this study are available from the corresponding author upon reasonable request.
